# Author Correction: Cytosolic actin isoforms form networks with different rheological properties that indicate specific biological function

**DOI:** 10.1038/s41467-024-45715-z

**Published:** 2024-02-09

**Authors:** Peter Nietmann, Kevin Kaub, Andrejus Suchenko, Susanne Stenz, Claas Warnecke, Mohan K. Balasubramanian, Andreas Janshoff

**Affiliations:** 1 Institute of Physical Chemistry, University of Goettingen, Tammannstr. 6, Göttingen, 37077 Germany; 2 Max Planck School Matter to Life, Max Planck Institute for Medical Research, Jahnstr. 29, Heidelberg, 69120 Germany; 3 Warwick Medical School, University of Warwick, Coventry, CV4 7AL UK

**Keywords:** Nanoscale biophysics, Biomaterials, Actin

Correction to: *Nature Communications* 10.1038/s41467-023-43653-w, published online 02 December 2023

In this article the funding from ‘Wellcome Trust WT101885MA and Biotechnology and Biological Sciences Research Council (BB/S003789/1)’ was omitted. The original article has been corrected.

