# Supplementary material for: Cytosolic actin isoforms form networks with different rheological properties that indicate specific biological function
Source: Nat Commun. 2023 Dec 2;14:7989. doi: 10.1038/s41467-023-43653-w (PMC10693642; doi:10.1038/s41467-023-43653-w)
Supplement: Supplementary file 1 — Supplementary Information [file 41467_2023_43653_MOESM1_ESM.pdf]

Supplementary Information

Mechanical properties of different actin isoforms vary - potential implications for biological function

Peter Nietmann[1] and Kevin Kaub[1,2], Andrejus Suchenko[3], Susanne Stenz[3], Claas Warnecke[3], Mohan K. Balasubramanian[3], Andreas Janshoff[1,2]

[1]Institute of Physical Chemistry, University of Goettingen,Tammannstr. 6,Göttingen,37077,Germany
[2]Max Planck School Matter to Life, Max Planck Institute for Medical Research, Jahnstr. 29, Heidelberg,69120, Germany
[3]Warwick Medical School, University of Warwick, Coventry, CV4 7AL,UK

Contents

1 Supplementary Methods 4
1.1 Filament length distribution and accumulated total filament length for each actin . . . 4
1.2 Determination of the plateau modulus G_0 . . . 5
1.3 Bulk rheology . . . 5
1.4 Two-particle microrheology . . . 5
1.5 Representation of standard deviations in logarithmic plots . . . 6

2 Supplementary Figures 7
2.1 Supplementary Figure 1 . . . 7
2.2 Supplementary Figure 2 . . . 8
2.3 Supplementary Figure 3 . . . 9
2.4 Supplementary Figure 4 . . . 10
2.5 Supplementary Figure 5 . . . 11
2.6 Supplementary Figure 6 . . . 12
2.7 Supplementary Figure 7 . . . 13
2.8 Supplementary Figure 8 . . . 14
2.9 Supplementary Figure 9 . . . 15
2.10 Supplementary Figure 10 . . . 16
2.11 Supplementary Figure 11 . . . 17
2.12 Supplementary Figure 12 . . . 18
2.13 Supplementary Figure 13 . . . 19
2.14 Supplementary Figure 14 . . . 20
2.15 Supplementary Figure 15 . . . 21
2.16 Supplementary Figure 16 . . . 22
2.17 Supplementary Figure 17 . . . 23
2.18 Supplementary Figure 18 . . . 24
2.19 Supplementary Figure 19 . . . 25

3 Supplementary Tables 26
3.1 Supplementary Table 1 . . . 26
3.2 Supplementary Table 2 . . . 26
3.3 Supplementary Table 3 . . . 27
3.4 Supplementary Table 4 . . . 27
3.5 Supplementary Table 5 . . . 27
3.6 Supplementary Table 6 . . . 27
3.7 Supplementary Table 7 . . . 28
3.8 Supplementary Table 8 . . . 28
3.9 Supplementary Table 9 . . . 28
3.10 Supplementary Table 10 . . . 29
3.11 Supplementary Table 11 . . . 29
3.12 Supplementary Table 12 . . . 29
3.13 Supplementary Tables 13 . . . 30
3.14 Supplementary Table 14 . . . 30
3.15 Supplementary Table 15 . . . 31
3.16 Supplementary Table 16 . . . 31
3.17 Supplementary Table 17 . . . 32
3.18 Supplementary Table 18 . . . 32
3.19 Supplementary Table 19 . . . 32

3.20 Supplementary Table 20 33
3.21 Supplementary Table 21 33
3.22 Supplementary Table 22 33
3.23 Supplementary Table 23 33
3.24 Supplementary Table 24 34
3.25 Supplementary Table 25 34
3.26 Supplementary Table 26 34
3.27 Supplementary Table 27 35
3.28 Supplementary Table 28 35
3.29 Supplementary Table 29 35
3.30 Supplementary Table 30 36
3.31 Supplementary Table 31 36
3.32 Supplementary Table 32 36
3.33 Supplementary Table 33 37
3.34 Supplementary Table 34 37
3.35 Supplementary Table 35 37
3.36 Supplementary Table 36 38
3.37 Supplementary Table 37 38
3.38 Supplementary Table 38 38

1 Supplementary Methods

1.1 Filament length distribution and accumulated total filament length for each actin

Supplementary Fig. 8 shows that a large part of the filaments counted in CLSM images is very small (smaller than 5 µm). However, the overwhelming majority of actin consists of monomers bound in long filaments (larger than 5 µm). Supplementary Figure 8 illustrates the cumulative total filament length (in orange) comprising all individual filament lengths.

Naturally, longer filaments exert a greater influence on network properties due to their higher actin content. To account for this, Burlacu and colleagues (*Am. J. Physiol. Cell Physiol.* 1992, **262(3)**, 569-577) introduced the weight average length L_w with

$$L_w = \frac{\sum_i N_i X_i^2}{\sum_i N_i X_i}.$$

Here the filament lengths are divided into intervals $I_i = i \times 0.5 + [-0.25, 0.25[$ $(i \in \mathbb{N})$ with mean $X_i = i \times 0.5 - 0.25$, both in dimension µm. The N_i represent the counts per interval I_i. From these results (Supplementary Table 1) we can safely assume that the overwhelming majority of actin consists of monomers bound in long filaments (larger than 5 µm).

Consequently, our filament length distributions align with the expected characteristics for F-actin solutions prepared using the same protocol. We also shortened F-actin filaments with the aid of gelsolin to reaffirm that our measurements are indeed focused on network properties. In Supplementary Fig. 10, the box plots illustrate how the plateau modulus (G' at 0.1 Hz) and fluidity (G''/G' at the G_0 position) of α-actin vary with different filament lengths: 1 µm, 5 µm, and 17.5 µm.

As expected, in line with both theoretical predictions (Maggs, *Phys. Rev. E* 1997, **55**, 7396) and previous experimental observations in the context of 1P microrheology experiments (Weitz and colleagues, *Phys. Rev. Lett.* 2006, **96**, 118104), our data demonstrates remarkably similar results for G_0 when comparing untreated F-actin networks to gelsolin-shortened networks with filament lengths of 17.5 µm and 5 µm. As filament length decreases down to 1 µm, the system's fluidity begins to rise, ultimately reaching its peak for the unpolymerized solution (G-actin).

Equally significant is the expected mesh size of our F-actin solutions. According to Schmidt's estimation (*Macromolecules* 1989, **22**, 3638):

$$\Longrightarrow \xi = \sqrt{\frac{3}{N_A \cdot c \cdot l_{\text{actin}}}} \approx 390\,\text{nm},$$

the mesh diameter is roughly five times smaller than the probe size (2 µm). This fulfills the condition for the probe to remain trapped within the F-actin mesh, while still placing it within the relevant length scales dictated by the network.
In addition, the plateau modulus G_0 remains roughly constant for beads whose size exceeds the meshsize of the surrounding actin network (Supplementary Fig. 5).

1.2 Determination of the plateau modulus G_0

Supplementary Table 3 shows G' for the three isoforms over a wide frequency range as well as for the minima of G'' and the tangent of the loss tangent $\tan\delta = \frac{G''}{G'}$, which can be utilized to determine G_0 [3]. 0.1 Hz is chosen for simplicity reasons and is congruent with convergence of 1P- and 2P microrheology data. There is no significant deviation in the results for β-actin and γ-actin compared to the other two common methods, which show minima between approximately 0.05 and 0.1 Hz in this concentration regime. The α-actin exhibits minor deviations within the error margin, which are significantly smaller than the variations observed between isoforms. Employing this fixed frequency offers improved signal-to-noise ratios, eliminating the need for smoothing or polynomial fitting of the more error-prone G'' data. Hence, the manipulation of data by smoothing is minimized. Fig. 2 in the manuscript displays the average mean square displacement of the pure isoforms, providing additional illustration that the variations in mechanical properties persist across the entire frequency spectrum.

1.3 Bulk rheology

We also conducted a comparative analysis of our holographic video particle tracking (hVPT) data with results obtained through various other methods. We have carried out the analysis solely for α-actin at different concentrations due to the substantial sample volume requirements for most of these techniques. These methods include active optical tweezers measurements (OT, microrheology), dynamic light scattering (DLS, microrheology), diffusing wave spectroscopy (DWS, macrorheology), and rheometer measurements (macrorheology), all performed under highly similar conditions to our hVPT setup.

Supplementary Figure 6 and Supplementary Table 4 illustrate a substantial concurrence among microrheological techniques, including VPTMR, OT, and DLS. Furthermore, there is a considerable alignment between microrheological methods and macrorheological techniques such as DWS and plate rheometry. Notably, the mean G_0 values obtained from macrorheological measurements are approximately 2-3 times larger as expected since 1P-MR does not capture long distance fluctuations.

1.4 Two-particle microrheology

In Supplementary Fig. 2 we show a comparison between one-particle microrheology (1P-MR) and two-particle microrheology (2P-MR) for α-actin and γ-actin. Our findings largely align with the results previously reported by Gardel *et al.* [1], where they observed that, for the same actin concentration we employed, the plateau moduli G_0 extracted from low frequencies are nearly indistinguishable. Regarding the frequency-dependent behavior and magnitude of the two-particle MSD (2P-MSD), it exhibits a qualitatively distinct profile when compared to the one-particle MSD (1P MSD) (Supplementary Fig. 2). 2P-MR displays enhanced viscoelastic relaxation at intermediate frequencies consistent with diffusion over the filament length L.[4] Specifically, at a time lag of $\tau = 0.1\,\text{s}$, the 2P MSD is nearly an order of magnitude smaller than the 1P MSD. Furthermore, the 2P MSD scales differently, with MSD $\propto \tau^{1/2}$, in contrast to the less pronounced change in the 1P-MSD with varying τ. At low frequencies, 1P-MR exhibits an elastic plateau in samples where $L > a$, attributable to the steric hindrance caused by filament entanglement at the entanglement length. We found that the plateau scales with the actin concentration as predicted for entangled F-actin networks (Fig. 2 in the main text). This same plateau is

eventually attained by 2P-MR, and we specifically selected a frequency of approximately 10 Hz for this purpose in order to extract the value of G_0.

It is noteworthy that, in accordance with the observations made by Gardel *et al.* and Liu *et al.*,[1, 4] the MSDs obtained from both 1P-MR and 2P-MR converge at low frequencies. However, it should be acknowledged that the data derived from 2P-MR exhibits poorer statistical reliability when compared to that of 1P-MR, particularly at the longer time scales we are interested in. Hence, we predominately used 1P MR in our study.

1.5 Representation of standard deviations in logarithmic plots

Plotting data points with their respective error bars in logarithmic plots leads to asymmetric bars with different lengths. For a better readability we chose to employ a standard procedure to represent the lower error bar:
Instead of considering the standard deviation Δx of a variable x as an absolute error, i.e. $x \pm \Delta x$, we plotted it (under the assumption of relatively small standard deviations) as relative error, i.e. the error Δz of the logarithmically represented variable $z = \log(x)$ is given by

$$\Delta z = \Delta(\log(x)) = \frac{1}{\log(10)} \frac{1}{x} \Delta x \approx 0.434 \frac{\Delta x}{x}.$$

2 Supplementary Figures

2.1 Supplementary Figure 1

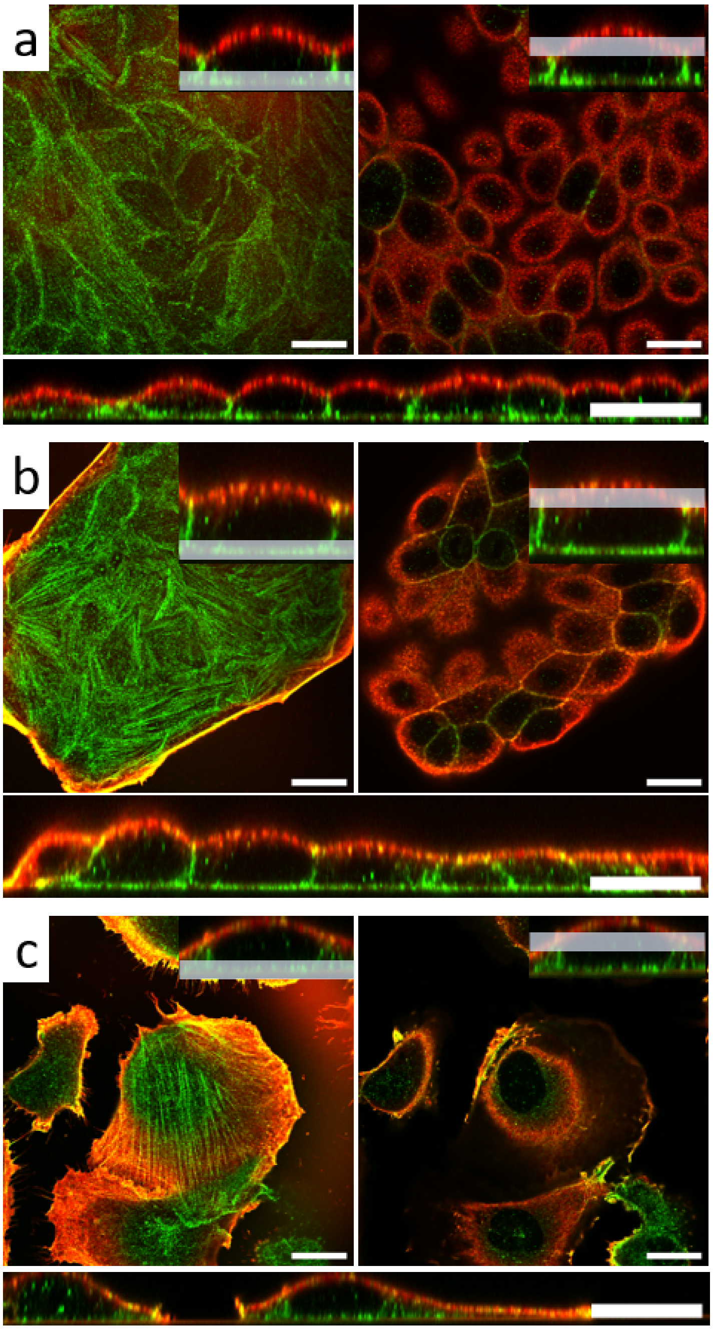

Supplementary Figure 1: Immunostaining of MDCK II and SK-OV-3. β-actin (green) and γ-actin (red) are locally segregated in MDCK II cells both in confluent monolayers (a) and in small clusters (b). β-actin is exclusively associated with basal stress-fibers (a,b, left panel), while γ-actin is predominately localized at the apical cortex (a,b, right panel). An orthogonal view (a,b, bottom panel) shows that the cell-cell-boundaries mainly consist of β-actin [6]. SK-OV-3 cells (c) show the same segregation pattern of actin isoforms. The staining was repeated with ≥ 5 samples. Sample statistics are provided in Sup. Tab. 20. (Scale bars: 20 μm

2.2 Supplementary Figure 2

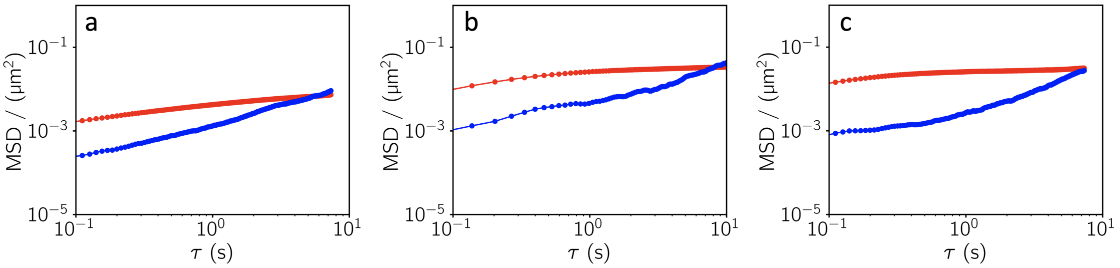

Supplementary Figure 2: Two-particle microrheology. Comparison of one-particle (red) and two-particle (blue) MSDs in 24 μM F-actin solution with particle radius 1 μm. Convergence is observed for lag times larger than 10 s. Measurement and sample statistics are provided in Sup. Tab. 19. The data underlying the figure is provided as Source Data.

2.3 Supplementary Figure 3

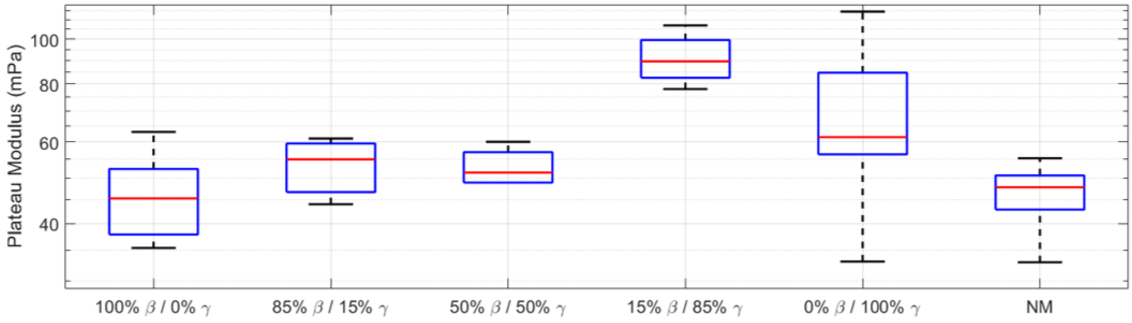

Supplementary Figure 3: Mechanical properties of networks formed by various mixtures of pure actin isoforms (β-actin and γ-actin) compared to NM-actin. The compositions (100/0, 85/15, 50/50, 15/85 0/100) are chosen for direct comparison with the NM-actin (85% β-actin and 15% γ-actin). Plateau moduli G_0 of the isoform mixtures and NM-actin are obtained from the purely elastic frequency regime of the viscoelastic spectra at a fixed frequency of 0.1 Hz. In each boxplot, the central thick black line represents the median, color shaded boxes represent the first and third quartiles (the 25th and 75th percentiles), and the whiskers extend no further than 1.5 times of the distance between the first and third quartiles. Measurement and sample statistics are provided in Sup. Tab. 21. The data underlying the figure is provided as Source Data.

2.4 Supplementary Figure 4

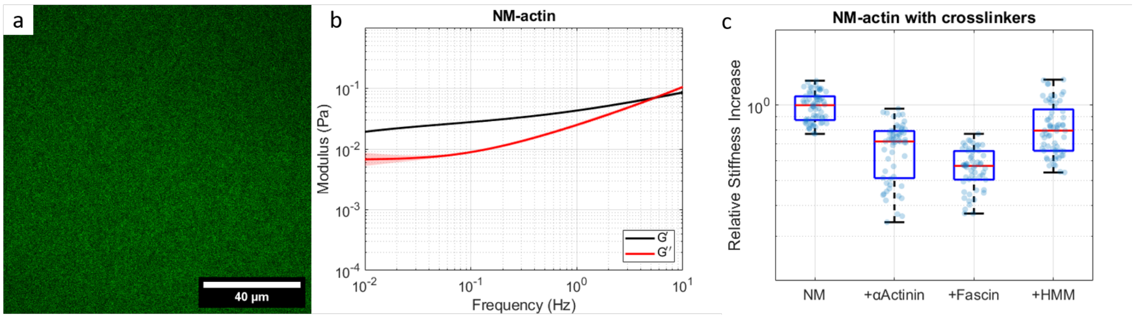

Supplementary Figure 4: Additional data for the commercially available NM-actin, a mix of 85 % β- and 15 % γ-actin. a: Shown is a CLSM image of an NM-actin network 12 µM. b: The viscoelastic spectrum of NM-actin networks at a concentration of 12 µM. c: Stiffness of NM-networks (12 µM) with α-actinin, fascin and HMM (each at 120 nM). In each boxplot, the central thick black line represents the median, color shaded boxes represent the first and third quartiles (the 25th and 75th percentiles), and the whiskers extend no further than 1.5 times of the distance between the first and third quartiles. Measurement and sample statistics are provided in Sup. Tab. 22. The data underlying the figure is provided as Source Data.

2.5 Supplementary Figure 5

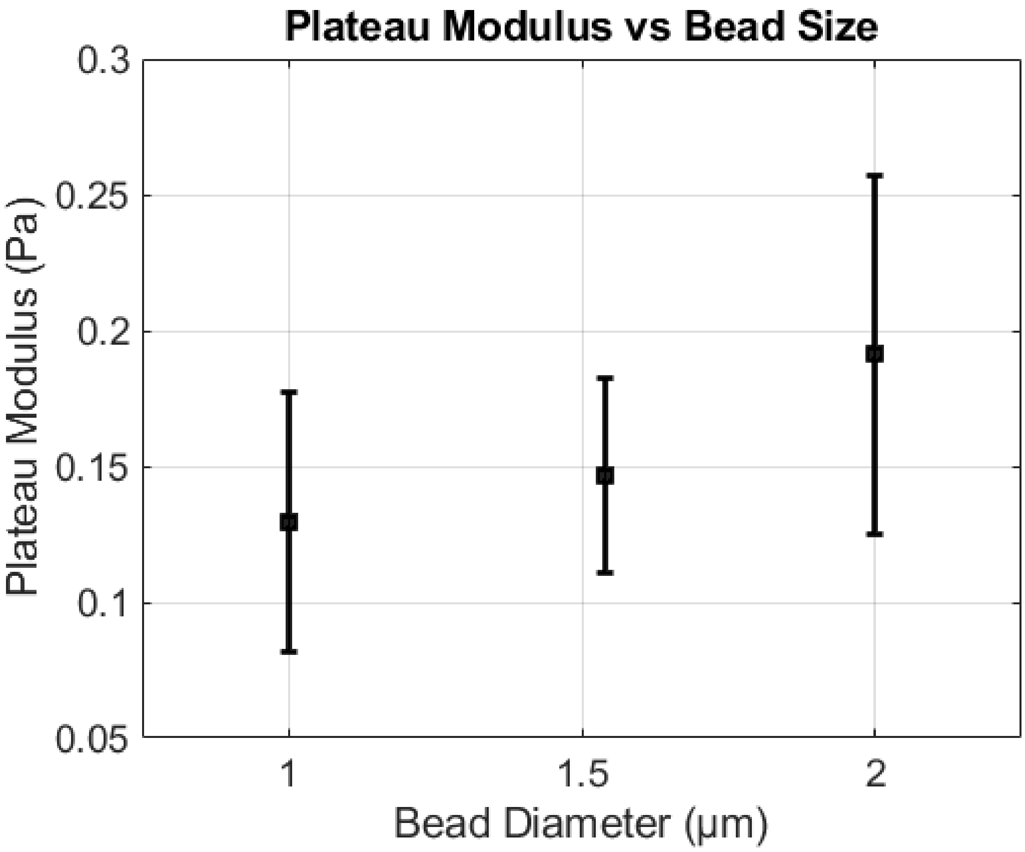

Supplementary Figure 5: Plateau moduli G_0 of α-networks formed at a concentration of 24 µM as a function of bead size. The plateau moduli on slightly increase with bead size. Data points are mean values and error bars are standard deviations. Measurement and sample statistics are provided in Sup. Tab. 23. The data underlying the figure is provided as Source Data.

2.6 Supplementary Figure 6

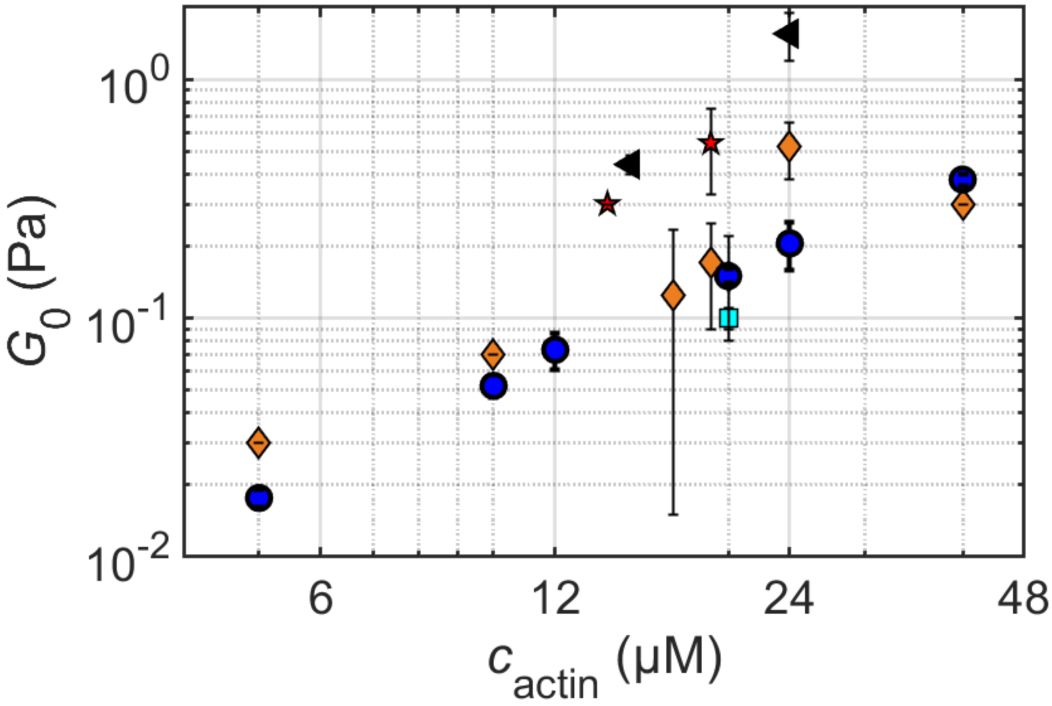

Supplementary Figure 6: An evaluation of various micro- and macrorheological techniques for assessing the mechanical characteristics of reconstituted F-actin networks is presented. The graph displays the average plateau moduli for α-actin networks across concentrations ranging from 5 to 40 μM. The primary method employed, holographic video particle tracking (1 particle), is represented by blue squares, while active optical tweezers (1 particle) are denoted by orange diamonds. Dynamic light scattering results are indicated by cyan squares, diffusing wave spectroscopy by black triangles, and measurements conducted with a rotating disc parallel plate rheometer are depicted as red stars. Data points are mean values and error bars are standard deviations. Measurement and sample statistics are provided in Sup. Tab. 24. The data underlying the figure is provided as Source Data.

2.7 Supplementary Figure 7

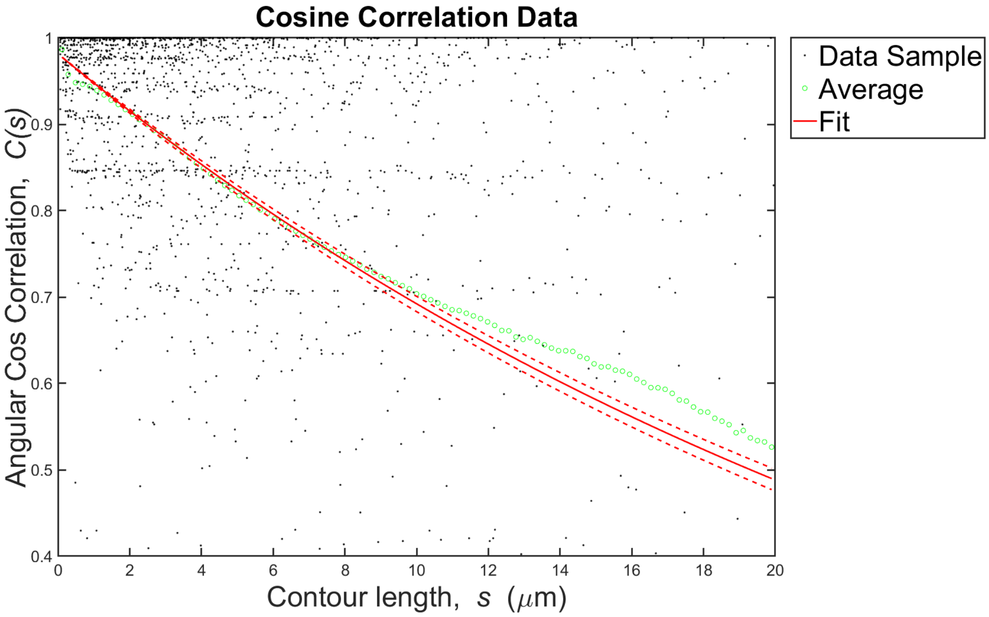

Supplementary Figure 7: Exemplary cosine correlation for α-actin (software: *Persistence*.[2]. Persistence lengths are determined with a fit (red) to the angular cosine correlation along skeletonized single filaments in solution. The average data (green) is fitted with $Ae^{2/2L_p}$ with the uncertainty of A as dashed red line. Micrographs of the filaments are taken with a CLSM and skeletonized with the ImageJ Plugin *Ridge Detection* (according to Steger 1998, https://imagej.net/plugins/ridge-detection).[5] Measurement and sample statistics are provided in Sup. Tab. 25. The data underlying the figure is provided as Source Data.

2.8 Supplementary Figure 8

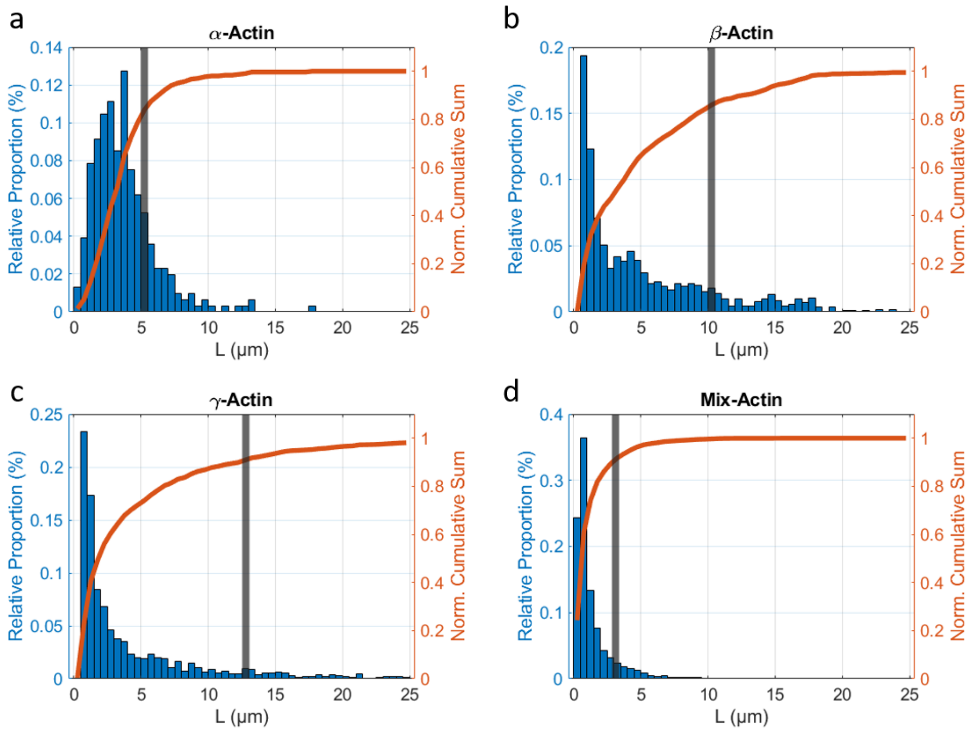

Supplementary Figure 8: Histograms of filament lengths for different actin isoforms and accumulated total filament length. The black vertical bar represents the weight average filament length. a: α-actin, b: β-actin, c: γ-actin, d: NM-actin. Measurement and sample statistics are provided in Sup. Tab. 26. The data underlying the figure is provided as Source Data.

2.9 Supplementary Figure 9

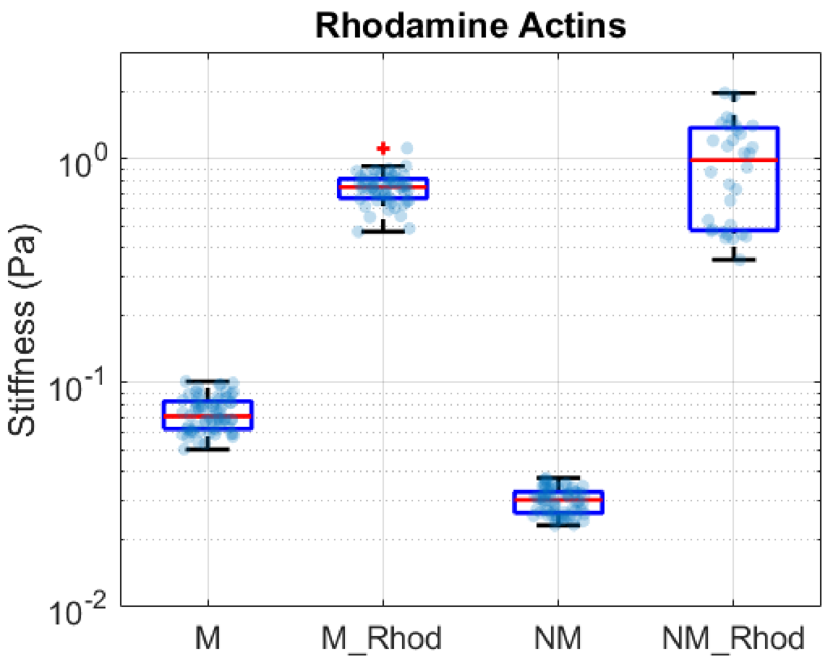

Supplementary Figure 9: Mechanical properties of networks formed by Rhodamine-labeled α-actin and NM-actin compared to unlabeled actins at a concentration of 12 μM. Plateau moduli G_0 of the two types are obtained from the purely elastic frequency regime of the viscoelastic spectra at a frequency of 0.1 Hz. In each boxplot, the central thick black line represents the median, color shaded boxes represent the first and third quartiles (the 25th and 75th percentiles), and the whiskers extend no further than 1.5 times of the distance between the first and third quartiles. Measurement and sample statistics are provided in Sup. Tab. 27. The data underlying the figure is provided as Source Data.

2.10 Supplementary Figure 10

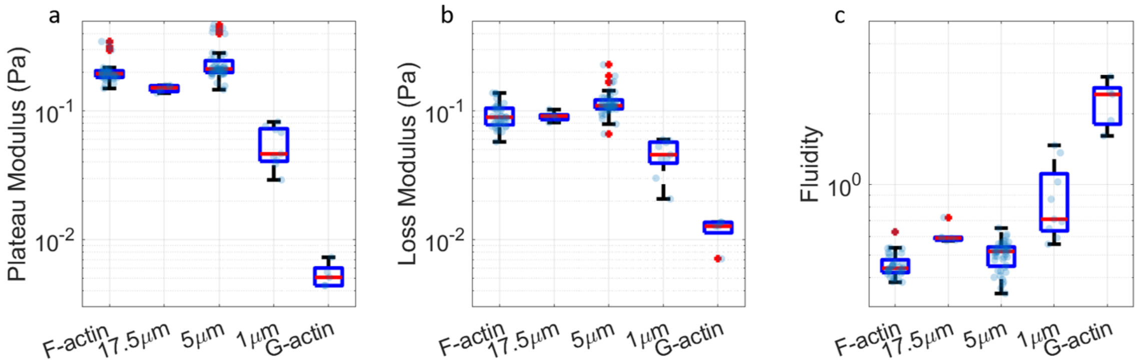

Supplementary Figure 10: Plateau moduli of α-actin obtained at a concentration of 24 μM as a function of contour length. Shown are microrheology measurements carried out with hVPT on networks with different filament lengths (an unmodified distribution, three gelsolin-controlled distributions with filament length averages of 17.5 μm, 5 μm and 1 μm as well as an unpolymerized G-actin solution. In each boxplot, the central thick black line represents the median, color shaded boxes represent the first and third quartiles (the 25th and 75th percentiles), and the whiskers extend no further than 1.5 times of the distance between the first and third quartiles. The first plot to the left (a) shows plateau moduli G_0, The second (b) in the center the loss moduli and the third one on the right (c), the fluidity (G''/G' at the position of G_0), respectively. Measurement and sample statistics are provided in Sup. Tab. 28. The data underlying the figure is provided as Source Data.

2.11 Supplementary Figure 11

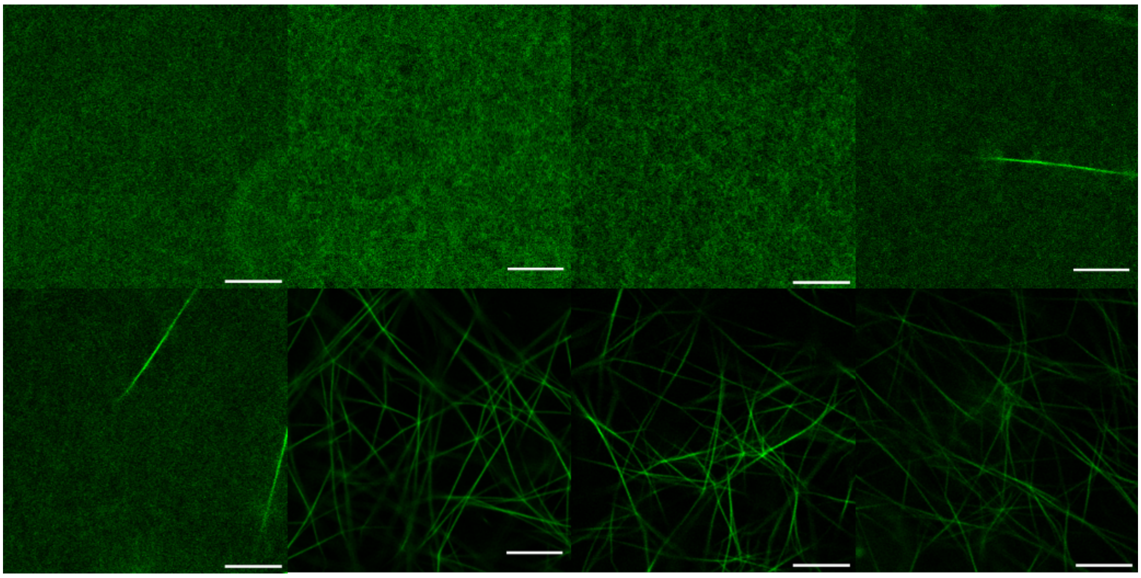

Supplementary Figure 11: Fluorescence microscopy images of the Mg^{2+} concentration-dependent bundling of α-actin networks. Shown are networks exposed to different concentrations of Mg^{2+}, starting with 5 mM on the upper left, then 10 and 15 and 20, 25 mM on the lower left, then 30, 35 and 40 mM . Bundling begins at 20 mM Mg^{2+}. At least 3 samples per Mg^{2+} concentration. Sample statistics are provided in Sup. Tab. 29. Scale bars: 10 µm.

2.12 Supplementary Figure 12

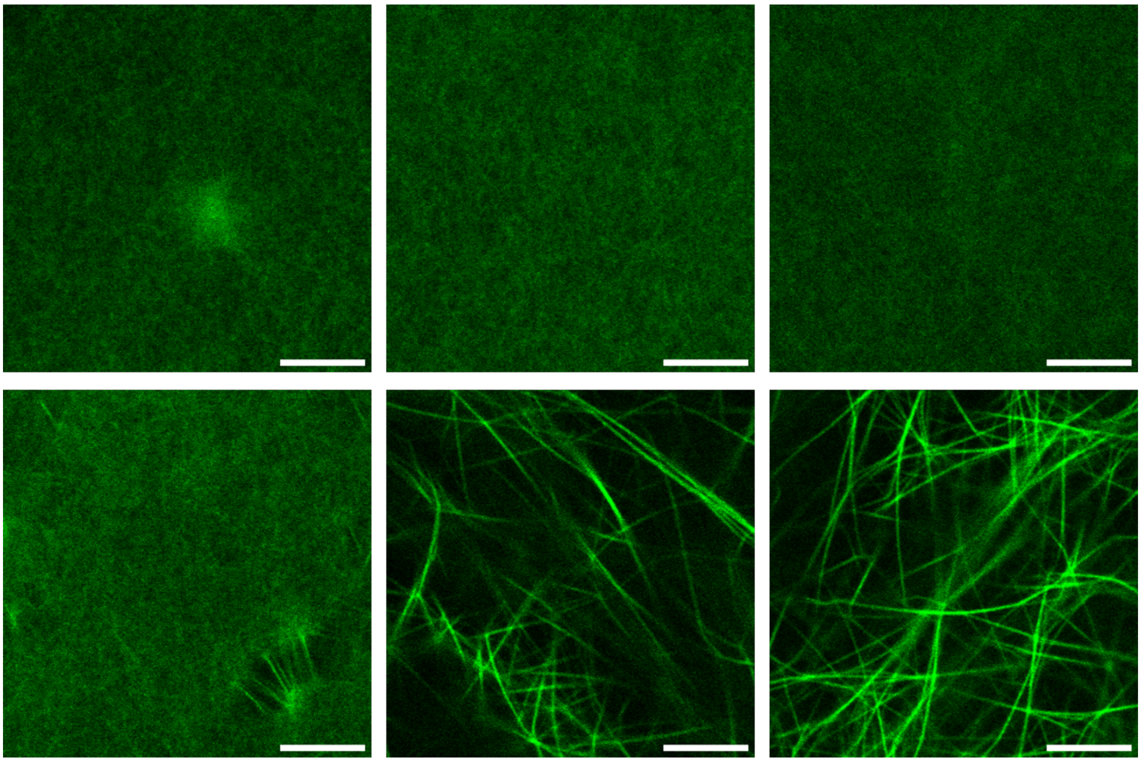

Supplementary Figure 12: Fluorescence microscopy images of the Mg^{2+} concentration-dependent bundling of β-actin networks. Shown are networks with different concentrations of Mg^{2+}, starting with 5 mM on the upper left, then 10 and 15, 20 mM on the lower left, then 25 and 30. Bundling begins at 20 mM Mg^{2+}. At least 3 samples per Mg^{2+} concentration. Sample statistics are provided in Sup. Tab. 30. Scale bars: 10 μm.

2.13 Supplementary Figure 13

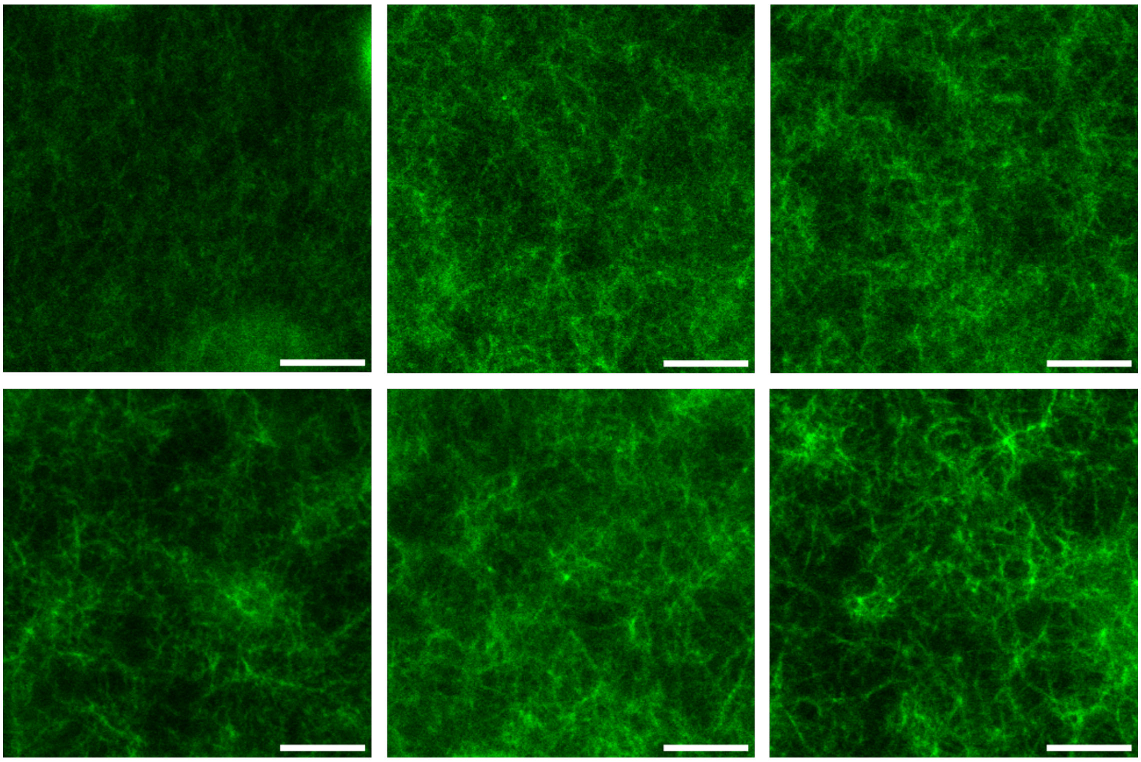

Supplementary Figure 13: Fluorescence microscopy images of the Mg^{2+} concentration-dependent bundling of γ-actin networks. Shown are networks with different concentrations of Mg^{2+}, starting with 5 mM on the upper left, then 10 and 15, 20 mM on the lower left, then 25 and 30. Bundling was not observed at these Mg^{2+} concentrations. At least 3 samples per Mg^{2+} concentration. Sample statistics are provided in Sup. Tab. 31. Scale bars: 10 μm.

2.14 Supplementary Figure 14

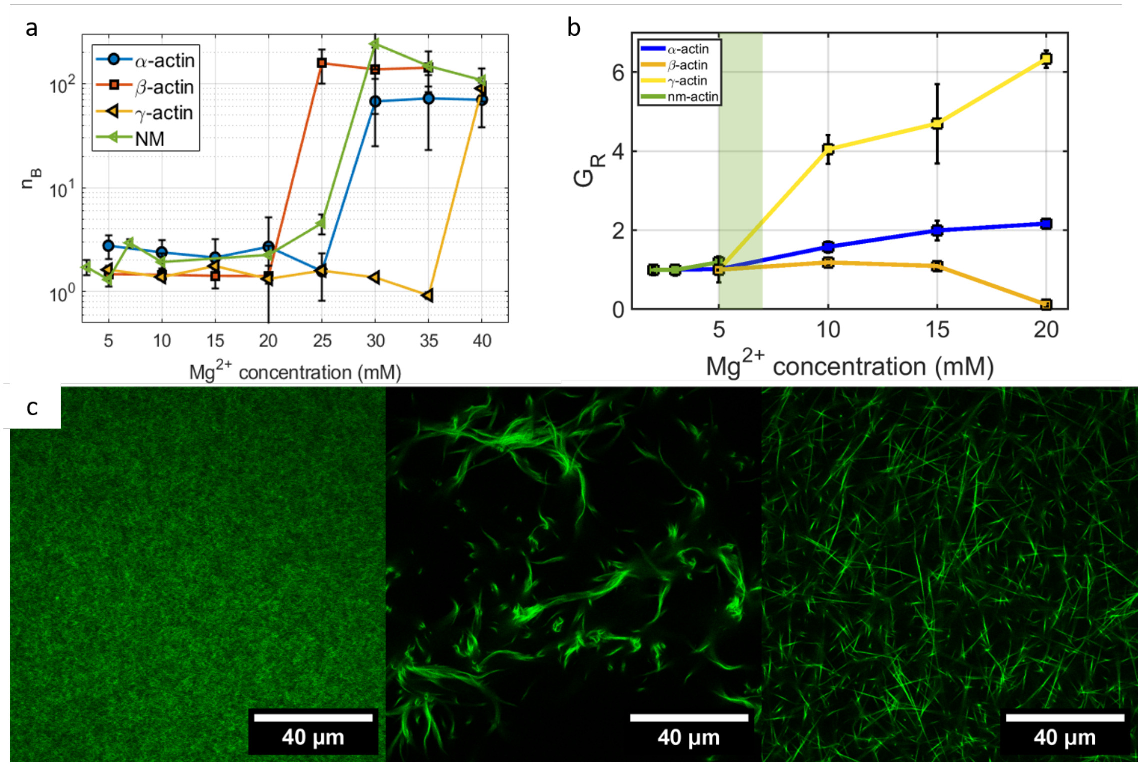

Supplementary Figure 14: Bundling parameter n_B (a), relative plateau modulus G_R (b) and representative CLSM images (c) of NM actin. For the CLSM images a concentration of 12 µM and magnesium concentrations of (from left to right) 5 mM, 15 mM and 40 mM. Note the emergence of an intermediate state of NM bundles at 15 mM Mg^{2+}. These intermediate bundles are present at concentrations between 10 mM and 25 mM. Data points are mean values and error bars are standard deviations. Measurement and sample statistics are provided in Sup. Tab. 32 and 33. The data underlying the figure is provided as Source Data.

2.15 Supplementary Figure 15

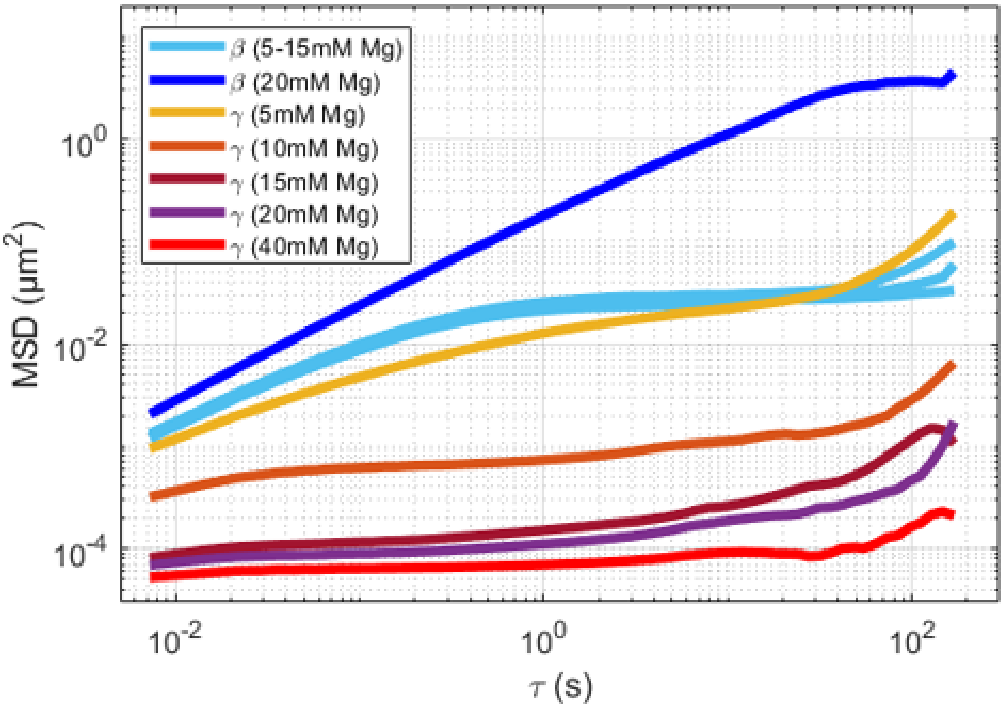

Supplementary Figure 15: MSDs of microparticles (size of 2 μm) entangled in β- and γ-actin networks (each at a concentration of 12 μM depending on increasing Mg^{2+} concentrations. For β-actin we don't have a noticable impact from 5 mM to 15 mM until bundling is initiated at 20 mM Mg^{2+}. At this treshold concentration the MSD drastically increases as the microparticles can move through the larger meshes of the bundled network without impedance. For γ-actin the MSD continuously decreases for Mg^{2+} concentrations starting from 5 mM upto 40 mM, indicative of a stiffening of the network. Data points are mean values. Compare Fig. 4c. Measurement and sample statistics are provided in Sup. Tab. 34. The data underlying the figure is provided as Source Data.

2.16 Supplementary Figure 16

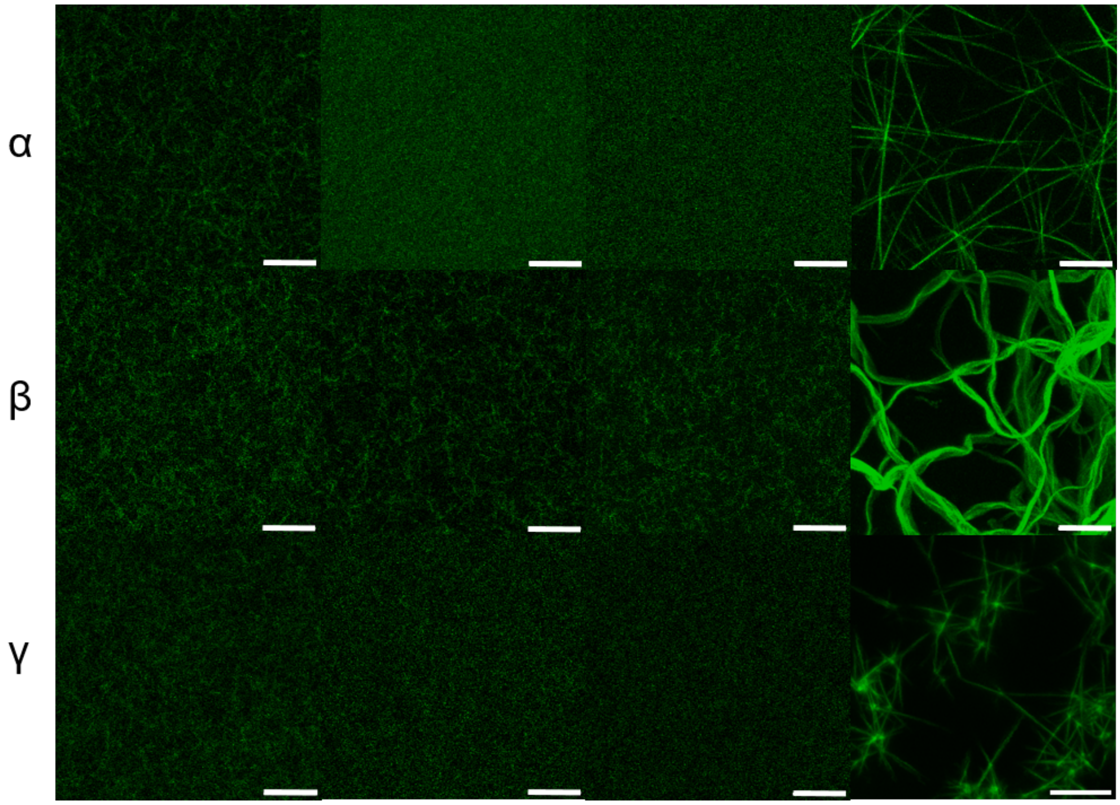

Supplementary Figure 16: Interaction/bundling of actin isoforms with Ca^{2+} ions. Shown are actin networks (α-, β- and γ-actin as indicated at 12 μM actin) at different concentrations of Ca^{2+} ions (0, 0.5, 1 and 5 mM from the left to the right panels; 0 μM networks are polymerized with 2 μM Mg^{2+} like usually, the others with 0 μM Mg^{2+}). Actin filament networks get bundled by Ca^{2+} ions in a process of counterion condensation like by Mg^{2+} ions. In contrary to Mg^{2+} induced bundling, isoform-specific bundling onset concentrations of the divalent cation can not be observed. α-, β- and γ-actin networks all show bundle formation at 10 mM Ca^{2+}. At least 2 samples per Mg^{2+} concentration. Sample statistics are provided in Sup. Tab. 35. Scale bars are 10 μm.

2.17 Supplementary Figure 17

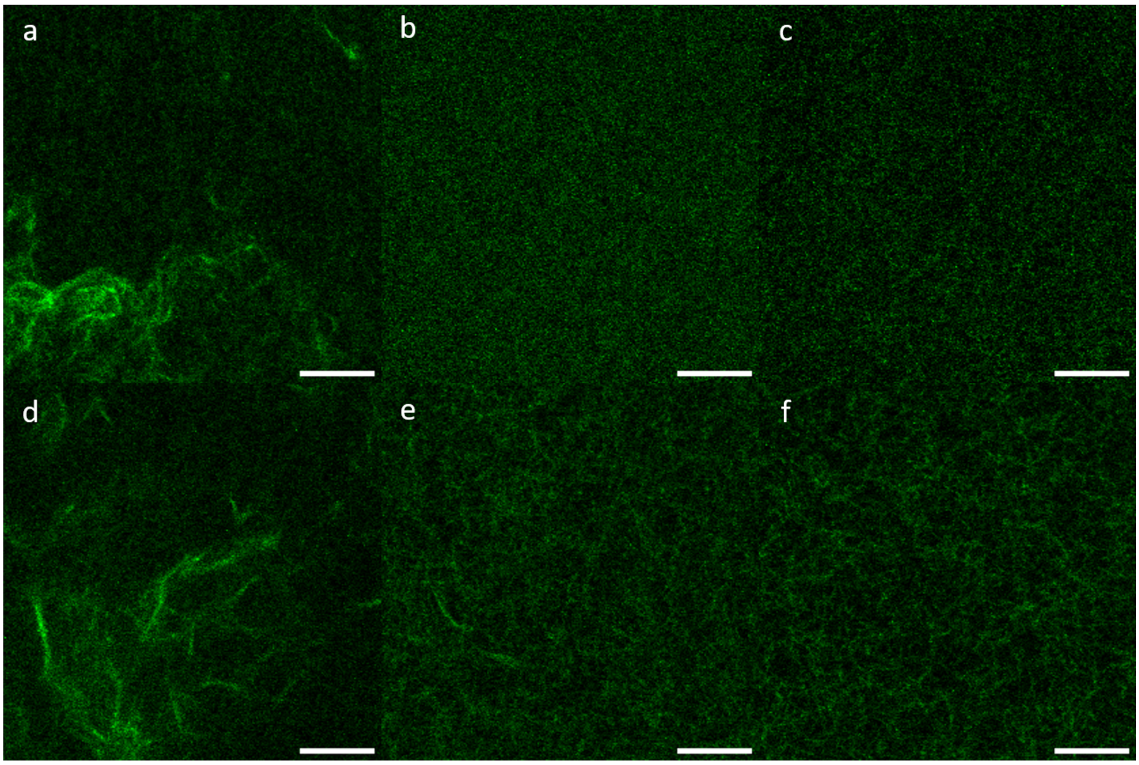

Supplementary Figure 17: Crosslinking of α-actin at elevated Mg^{2+} concentrations. No bundling is observable at the chosen actin- and crosslinker concentrations (12 µM actin, 120 nM crosslinkers and 2 mM Mg^{2+}). It is expected that bundling occurs at elevated concentrations of actin and (bundling-) crosslinkers. Shown are α-actin networks crosslinked with different actin-binding proteins (a and d: α-actinin, b and e: fascin and c and f: HMM) at different Mg^{2+} concentrations (upper panels are at 7.5 mM Mg^{2+}, lower panels at 15 mM Mg^{2+}). At least 2 samples per Mg^{2+} concentration. Sample statistics are provided in Sup. Tab. 36. Scale bars are 10 µm.

2.18 Supplementary Figure 18

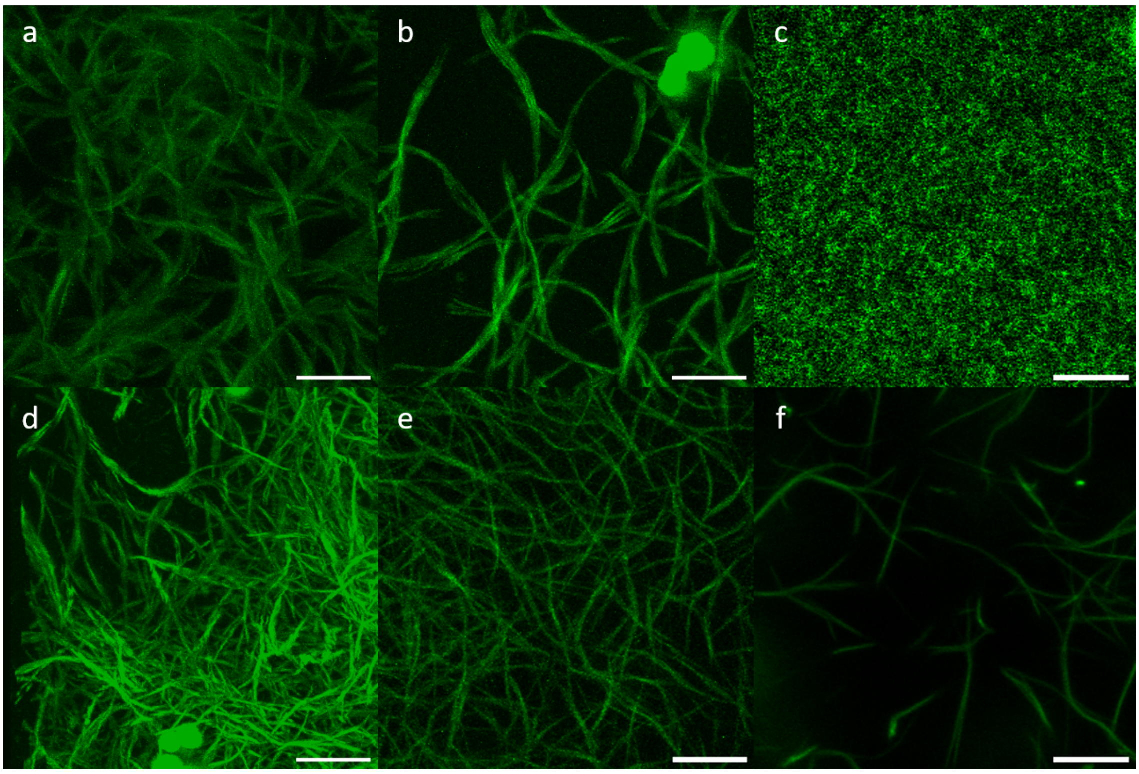

Supplementary Figure 18: Crosslinking of β-actin at elevated Mg^{2+} concentrations. No bundling is observable at the chosen actin- and crosslinker concentrations (12 µM actin, 120 nM crosslinkers and 2 mM Mg^{2+}). Shown are β-actin networks crosslinked with different crosslinkers (a and d: α-actinin, b and e: fascin and c and f: HMM) at different Mg^{2+} concentrations (upper panels are at 7.5 mM Mg^{2+}, lower panels at 15 mM Mg^{2+}). At least 2 samples per Mg^{2+} concentration. Sample statistics are provided in Sup. Tab. 37. Scale bars are 10 µm.

2.19 Supplementary Figure 19

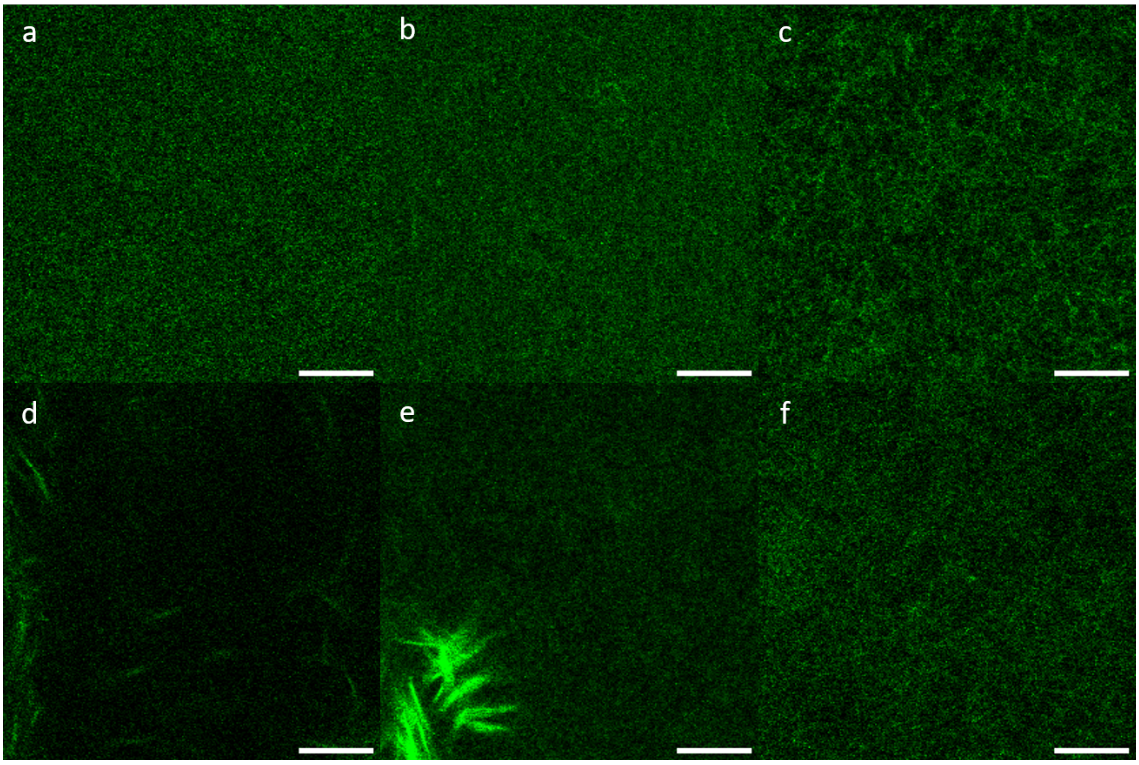

Supplementary Figure 19: Crosslinking of γ-actin at elevated Mg^{2+} concentrations. No bundling is observable at the chosen actin- and crosslinker concentrations (12 μM actin, 120 nM crosslinkers and 2 mM Mg^{2+}). Shown are γ-actin networks crosslinked with different crosslinkers (a and d: α-actinin, b and e: fascin and c and f: HMM) at different Mg^{2+} concentrations (upper panels are at 7.5 mM Mg^{2+}, lower panels at 15 mM Mg^{2+}). At least 2 samples per Mg^{2+} concentration. Sample statistics are provided in Sup. Tab. 38. Scale bars are 10 μm.

3 Supplementary Tables

3.1 Supplementary Table 1

Supplementary Table 1: Storage moduli of the various isoforms at different frequencies. Compare Supplementary Fig. 20 for measurement statistics.

Frequency (Hz)	α-G'(mPa)	β-G'(mPa)	γ-G'(mPa)
1	115 ± 20	9 ± 1	40 ± 3
0.5	100 ± 16	8 ± 1	38 ± 3
**0.1**	$\mathbf{79 \pm 11}$	$\mathbf{7 \pm 1}$	$\mathbf{39 \pm 3}$
0.05	76 ± 10	7 ± 1	39 ± 3
0.01	71 ± 10	7 ± 1	40 ± 3
min G''	75 ± 10	7 ± 1	39 ± 3
min tan δ	75 ± 10	7 ± 1	39 ± 3

3.2 Supplementary Table 2

Supplementary Table 2: Comparison of different rheological methods. Shown are mean shear moduli $\bar{G}_0$ of α-actin at a concentration of 20 μM obtained from Supplementary Fig. 6. Standard deviation from multiple samples and measurements. Compare Supplementary Fig. 6

c_{actin} (μM)	Method	$\bar{G}_0$ (Pa)
20	hVPT	0.15 ± 0.01
17, 19 and 20	OT	0.16 ± 0.07
20	DLS	0.10 ± 0.01
15	DWS	0.44 ± 0.04
19	Rheometer	0.55 ± 0.23

3.3 Supplementary Table 3

Actin (label and analysis method)	l_p [µm]
α(phalloidin label)	15.43 ± 0.43
β(phalloidin label)	16.36 ± 0.46
γ(phalloidin label)	16.77 ± 0.41
NM (phalloidin label)	15.49 ± 0.42
NM (rhodamine label)	10.27 ± 0.42
β(phalloidin label, pre-labled, on Poly-L-Lysine surface)	4.72± 0.19
β(phalloidin label, post-labled on Poly-L-Lysine surface)	3.10± 0.09
γ(phalloidin label, post-labled on Poly-L-Lysine surface)	2.73± 0.08

Supplementary Table 3: Persistence lengths of isoforms obtained from cosine correlation. A small portion of fully polymerized and phalloidin-labeled actin is added into a larger volume of buffer and the filaments were allowed to equilibrate over a long period of time. Afterwards the liquid with the single filaments was cast into glass chambers (depth of 2 µm) to reduce undulations to 2 dimensions. For comparison, rhodamine labled NM-actin was additionally analyzed together with β- and γ-actin pre- and post-labled on on Poly-L-Lysine coated surfaces. Compare materials and methods. Persistence length obtained from ≥ 1000 filaments (See Sup. Tab. 26).

3.4 Supplementary Table 4

Supplementary Table 4: Weight average length L_w and the number average length $L_n = (\sum N_i X_i)/(\sum N_i)$ with corresponding standard deviations, as defined in Burlacu *et al.* (1992). The number of filaments is shown in Sup. Tab. 26

	α	β	γ	mix
L_w (µm)	5.2 ± 3.0	10.3 ± 6.7	12.8 ± 10.9	3.1 ± 2.5
L_n (µm)	3.7 ± 2.6	5.1 ± 7.2	4.6 ± 10.2	1.4 ± 2.1

3.5 Supplementary Table 5

Supplementary Table 5: Measurement statistics for Fig. 1a. Compare Sup. Tab. 20.

CLSM, Immunostaining		
plot	images	samples
MDCK-II cluster	5	1

3.6 Supplementary Table 6

Supplementary Table 6: Measurement statistics for Fig. 1f.

CLSM, Network structure		
plot	images	samples
γ(12 µM) Mg^{2+} (30 mM)	9	2

3.7 Supplementary Table 7

Supplementary Table 7: Measurement statistics for Fig. 2.

hVPT, Network mechanics of isoforms			
plot	samples	measurements	probes
$\alpha(5\,\mu M)$	1	3	50
$\alpha(6\,\mu M)$	4	32	639
$\alpha(10\,\mu M)$	2	5	70
$\alpha(12\,\mu M)$	8	64	1127
$\alpha(20\,\mu M)$	1	3	46
$\alpha(24\,\mu M)$	9	42	610
$\beta(3\,\mu M)$	2	5	54
$\beta(6\,\mu M)$	1	3	74
$\beta(12\,\mu M)$	10	76	1277
$\beta(24\,\mu M)$	7	41	604
$\gamma(6\,\mu M)$	4	16	372
$\gamma(12\,\mu M)$	12	96	1500
$\gamma(18\,\mu M)$	4	12	192
$\gamma(21.4\,\mu M)$	1	2	16
$\gamma(24\,\mu M)$	7	41	604

3.8 Supplementary Table 8

Supplementary Table 8: p-values for Fig. 2b calculated via Wilcoxon rank sum test.

hVPT, Network mechanics of isoforms				
	$\alpha(12\,\mu M)$	$\beta(12\,\mu M)$	$\gamma(12\,\mu M)$	NM $(12\,\mu M)$
$\alpha(12\,\mu M)$	-	$7.8 \cdot 10^{-25}$	$4.6 \cdot 10^{-26}$	$1.7 \cdot 10^{-22}$
$\beta(12\,\mu M)$	-	-	$3.8 \cdot 10^{-30}$	$7.8 \cdot 10^{-25}$
$\gamma(12\,\mu M)$	-	-	-	$1.3 \cdot 10^{-15}$

3.9 Supplementary Table 9

Supplementary Table 9: Measurement statistics for Fig. 3.

CLSM, Interaction with Myosin		
plot	images	samples
α	30	4
β	20	3
γ	13	2
NM	11	1

3.10 Supplementary Table 10

Supplementary Table 10: p-values for Fig. 3b calculated via Wilcoxon rank sum test.

CLSM, aster area				
	α	β	γ	NM
α	-	0	$1.1 \cdot 10^{-17}$	$3.0 \cdot 10^{-20}$
β	-	-	0	0
γ	-	-	-	0.49

3.11 Supplementary Table 11

Supplementary Table 11: p-values for Fig. 3c calculated via Wilcoxon rank sum test.

CLSM, aster area fraction				
	α	β	γ	NM
α	-	0.0021	0.9445	0.0014
β	-	-	$9.12 \cdot 10^{-5}$	$6.12 \cdot 10^{-6}$
γ	-	-	-	$9.92 \cdot 10^{-4}$

3.12 Supplementary Table 12

Supplementary Table 12: p-values for Fig. 3d calculated via Wilcoxon rank sum test.

CLSM, aster distance				
	α	β	γ	NM
α	-	0	$7.5 \cdot 10^{-158}$	$3.8 \cdot 10^{-119}$
β	-	-	0	0
γ	-	-	-	0.4075

3.13 Supplementary Tables 13

Supplementary Table 13: Measurement statistics for Fig. 4a.

CLSM, Bundle number		
plot	images	samples
α12Mg5	21	3
α12Mg10	22	3
α12Mg15	24	3
α12Mg20	26	3
α12Mg25	18	2
α12Mg30	16	2
α12Mg35	18	2
α12Mg40	20	2
β12Mg5	14	4
β12Mg10	13	4
β12Mg15	13	4
β12Mg20	15	4
β12Mg25	11	3
β12Mg30	11	3
β12Mg35	9	2
γ12Mg5	10	3
γ12Mg10	12	3
γ12Mg15	10	3
γ12Mg20	13	3
γ12Mg25	14	3
γ12Mg30	13	3
γ12Mg35	6	1
γ12Mg40	5	1

3.14 Supplementary Table 14

Supplementary Table 14: Measurement statistics for Fig. 4b.

CLSM, Isoform networks and Mg^{2+}		
plot	images	samples
All isoforms	See Sup. Tab. 29,30 and 31	See Sup. Tab. 29,30 and 31

3.15 Supplementary Table 15

Supplementary Table 15: Measurement statistics for Fig. 4c.

hVPT, Isoform networks and Mg^{2+}			
plot	samples	measurements	probes
α(12 μM) Mg^{2+} (2 mM)	8	64	1127
α(12 μM) Mg^{2+} (5 mM)	3	6	133
α(12 μM) Mg^{2+} (10 mM)	3	6	138
α(12 μM) Mg^{2+} (15 mM)	3	6	138
α(12 μM) Mg^{2+} (20 mM)	3	6	138
β(12 μM) Mg^{2+} (5 mM)	3	3	59
β(12 μM) Mg^{2+} (10 mM)	2	3	61
β(12 μM) Mg^{2+} (15 mM)	2	3	66
β(12 μM) Mg^{2+} (20 mM)	2	3	53
γ(12 μM) Mg^{2+} (5 mM)	4	8	152
γ(12 μM) Mg^{2+} (10 mM)	4	8	150
γ(12 μM) Mg^{2+} (15 mM)	4	7	134
γ(12 μM) Mg^{2+} (20 mM)	4	6	128

3.16 Supplementary Table 16

Supplementary Table 16: Measurement statistics for Fig. 4d.

CLSM, Isoform networks and Ca^{2+}			
plot	samples	measurements	probes
α12Mg2	8	64	1127
α12Ca0.5	3	12	228
α12Ca1	2	8	106
α12Ca5	3	12	228
β12Mg2	10	76	1277
β12Ca0.5	3	8	184
β12Ca1	3	9	216
β12Ca5	3	9	216
γ12Mg2	3	6	96
γ12Ca0.5	3	9	120
γ12Ca1	3	9	129
γ12Ca5	3	9	129

3.17 Supplementary Table 17

Supplementary Table 17: Measurement statistics for Fig. 5.

hVPT, Crosslinkers			
plot	samples	measurements	probes
α(12 μM)	8	64	1127
α12Mg15	2	8	128
α12αA100	10	82	1309
α12Fa100	8	64	1078
α12HMM100	8	64	1030
β(12 μM)	10	76	1277
β12Mg15	2	12	264
β12αA100	8	64	626
β12Fa100	8	63	573
β12HMM100	11	88	1386
γ(12 μM)	12	96	1500
γ12Mg15	2	10	284
γ12αA100	10	80	817
γ12Fa100	10	74	813
γ12HMM100	10	81	1043

3.18 Supplementary Table 18

Supplementary Table 18: p-values for Fig. 5 calculated via Wilcoxon rank sum test.

hVPT, Crosslinkers				
	+15 mM Mg^{2+}	+120 nM α-actinin	+120 nM fascin	+120 nM HMM
α	0.2705	$1.4 \cdot 10^{-7}$	$2.2 \cdot 10^{-6}$	$1.7 \cdot 10^{-22}$
β	0.9334	$1.4 \cdot 10^{-18}$	0.5733	$5.3 \cdot 10^{-29}$
γ	$2.2 \cdot 10^{-7}$	$2.4 \cdot 10^{-6}$	0.0228	$2.4 \cdot 10^{-30}$

3.19 Supplementary Table 19

Supplementary Table 19: Measurement statistics for Sup Fig. 1.

VPT, 2PMR			
plot	samples	measurements	probes
α(12 μM)	1	18	>100
β(12 μM)	1	41	>100
γ(12 μM)	1	17	>100

3.20 Supplementary Table 20

Supplementary Table 20: Measurement statistics for Sup. Fig. 2.

CLSM, Immunostaining		
plot	images	samples
SK-OV-3 single cells	8	1
MDCK-II cluster	5	1
MDCK-II monolayers	19	3

3.21 Supplementary Table 21

Supplementary Table 21: Measurement statistics for Sup. Fig. 3.

hVPT, Mixtures of Isoforms			
plot	samples	measurements	probes
β(12 μM)	10	76	1277
85% β-actin and 15% γ-actin	2	3	54
50% β-actin and 50% γ-actin	2	4	39
15% β-actin and 85% γ-actin	2	4	69
γ(12 μM)	12	96	1500
NM-actin	8	64	1169

3.22 Supplementary Table 22

Supplementary Table 22: Measurement statistics for Sup. Fig. 4.

hVPT/CLSM, NM-actin			
plot	samples	measurements	probes
NM(12 μM)	8	64	1169
NM12αA100	7	55	702
NM12Fa100	7	55	885
NM12HMM100	8	66	1066
plot	images	samples	
NM(12 μM)	3	2	

3.23 Supplementary Table 23

Supplementary Table 23: Measurement statistics for Sup. Fig. 5.

hVPT, Influence of particle size			
plot	samples	measurements	probes
M12(1 μm)	2	10	160
M12(1.5 μm)	2	10	246
M12(2 μm)	9	42	610

3.24 Supplementary Table 24

Supplementary Table 24: Measurement statistics for Sup. Fig. 6.

R/MR, Different methods			
plot	samples	measurements	probes
hVPT α(5 μM)	1	3	50
hVPT α(6 μM)	4	32	639
hVPT α(10 μM)	2	5	70
hVPT α(12 μM)	8	64	1127
hVPT α(20 μM)	1	3	46
hVPT α(24 μM)	9	42	610
hVPT α(40 μM)	1	3	76
OT α(5 μM)	1	1	17
OT α(10 μM)	3	11	40
OT α(17 μM)	4	5	27
OT α(19 μM)	4	22	109
OT α(20 μM)	1	1	5
OT α(24 μM)	12	13	98
OT α(40 μM)	1	1	8
DLS α(20 μM)	2	9	$>10^3$
DWS α(16 μM)	2	29	$>10^3$
DWS α(24 μM)	2	11	$>10^3$
Rheometer α(14 μM)	1	1	1
Rheometer α(19 μM)	11	11	11

3.25 Supplementary Table 25

Supplementary Table 25: Measurement statistics for Sup. Fig. 7.

CLSM, Cos.Corr. α			
plot	images	samples	filaments
α	>300	10	2035

3.26 Supplementary Table 26

Supplementary Table 26: Measurement statistics for Sup. Fig. 8.

CLSM, Filament l_c and l_p			
plot	images	samples	filaments
α	>300	10	2035
β	>300	10	1156
γ	>300	10	1636
NM	>300	10	5672

3.27 Supplementary Table 27

Supplementary Table 27: Measurement statistics for Sup. Fig. 9.

hVPT, Rhodamine labeled a			
plot	samples	measurements	probes
NM(12 μM)	8	64	1169
NM12Rhod	2	16	208
α(12 μM)	8	64	1127
α12Rhod	4	32	343

3.28 Supplementary Table 28

Supplementary Table 28: Measurement statistics for Sup. Fig. 10.

hVPT, Contour length and Fluidity			
plot	samples	measurements	probes
α(24 μM) (hVPT)	9	42	610
α12Lc17.5 (24 μM) (hVPT)	2	6	75
α12Lc5 (24 μM) (hVPT)	10	44	652
α12Lc1 (24 μM) (hVPT)	3	9	144
α12unpol (24 μM) (OT)	1	5	5

3.29 Supplementary Table 29

Supplementary Table 29: Measurement statistics for Sup. Fig. 11.

CLSM, α-actin and Mg^{2+}		
plot	images	samples
α12Mg2	8	2
α12Mg5	31	5
α12Mg10	31	4
α12Mg15	40	6
α12Mg20	31	5
α12Mg25	14	3
α12Mg30	18	4
α12Mg35	19	3
α12Mg40	16	5

3.30 Supplementary Table 30

Supplementary Table 30: Measurement statistics for Sup. Fig. 12.

CLSM, β-actin and Mg^{2+}		
plot	images	samples
β12Mg2	10	2
β12Mg5	14	4
β12Mg10	13	4
β12Mg15	13	4
β12Mg20	15	4
β12Mg25	11	3
β12Mg30	11	3
β12Mg35	9	2

3.31 Supplementary Table 31

Supplementary Table 31: Measurement statistics for Sup. Fig. 13.

CLSM, γ-actin and Mg^{2+}		
plot	images	samples
γ12Mg2	10	2
γ12Mg5	10	3
γ12Mg10	12	3
γ12Mg15	10	3
γ12Mg20	13	3
γ12Mg25	14	3
γ12Mg30	13	3
γ12Mg35	6	1
γ12Mg40	5	1

3.32 Supplementary Table 32

Supplementary Table 32: Measurement statistics for Sup. Fig. 14a,c.

CLSM, NM-actin bundling		
plot	images	samples
NM12Mg2	3	2
NM12Mg3	22	4
NM12Mg5	31	5
NM12Mg7	19	4
NM12Mg10	12	3
NM12Mg15	5	2
NM12Mg20	14	3
NM12Mg25	15	3
NM12Mg30	2	2
NM12Mg35	10	2
NM12Mg40	1	1

3.33 Supplementary Table 33

Supplementary Table 33: Measurement statistics for Sup. Fig. 14b. Compare Sup. Fig. 15.

hVPT, NM-actin and Mg^{2+}			
plot	samples	measurements	probes
NM12Mg2	4	24	542
NM12Mg3	4	24	540
NM12Mg5	4	24	537
NM12Mg7	4	24	547

3.34 Supplementary Table 34

Supplementary Table 34: Measurement statistics for Sup. Fig. 15.

hVPT, MSD Mg^{2+}			
plot	samples	measurements	probes
β12Mg5	3	3	59
β12Mg10	2	3	61
β12Mg15	2	3	66
β12Mg20	2	3	53
γ12Mg5	4	8	152
γ12Mg10	4	8	150
γ12Mg15	4	7	134
γ12Mg20	4	6	128
γ12Mg40	2	5	109

3.35 Supplementary Table 35

Supplementary Table 35: Measurement statistics for Sup. Fig. 16.

CLSM, Isoforms and Calcium		
plot	images	samples
α12Mg2	8	2
α12Ca0.5	19	4
α12Ca1	18	4
α12Ca5	24	4
β12Mg2	10	2
β12Ca0.5	12	3
β12Ca1	14	4
β12Ca5	15	4
γ12Mg2	10	2
γ12Ca0.5	14	4
γ12Ca1	16	4
γ12Ca5	15	4

3.36 Supplementary Table 36

Supplementary Table 36: Measurement statistics for Sup. Fig. 17.

CLSM, bundling of α-actin by Mg^{2+}		
plot	images	samples
α12aA100Mg7.5	20	3
α12aA100Mg15	21	3
α12Fa100Mg7.5	20	3
α12Fa100Mg15	24	3
α12HMM100Mg7.5	27	3
α12HMM100Mg15	13	2

3.37 Supplementary Table 37

Supplementary Table 37: Measurement statistics for Sup. Fig. 18.

CLSM, bundling of β-actin by Mg^{2+}		
plot	images	samples
β12aA100Mg7.5	19	2
β12aA100Mg15	17	2
β12Fa100Mg7.5	18	2
β12Fa100Mg15	18	2
β12HMM100Mg7.5	18	2
β12HMM100Mg15	20	2

3.38 Supplementary Table 38

Supplementary Table 38: Measurement statistics for Sup. Fig. 19.

CLSM, bundling of γ-actin by Mg^{2+}		
plot	images	samples
γ12aA100Mg7.5	20	2
γ12aA100Mg15	24	2
γ12Fa100Mg7.5	20	2
γ12Fa100Mg15	23	2
γ12HMM100Mg7.5	17	2
γ12HMM100Mg15	15	2

References

[1] M. L. Gardel et al. “Microrheology of Entangled F-Actin Solutions”. In: *Physical Review Letters* 91.15 (Oct. 2003). DOI: 10.1103/physrevlett.91.158302. URL: https://doi.org/10.1103%2Fphysrevlett.91.158302.

[2] John S Graham et al. “Multi-platform compatible software for analysis of polymer bending mechanics”. In: *PloS one* 9.4 (2014), e94766.

[3] Chenyang Liu et al. “Evaluation of different methods for the determination of the plateau modulus and the entanglement molecular weight”. In: *Polymer* 47.13 (2006), pp. 4461–4479.

[4] J. Liu et al. “Microrheology Probes Length Scale Dependent Rheology”. In: *Physical Review Letters* 96.11 (Mar. 2006). DOI: 10.1103/physrevlett.96.118104. URL: https://doi.org/10.1103/physrevlett.96.118104.

[5] Carsten Steger. “An unbiased detector of curvilinear structures”. In: *IEEE Transactions on pattern analysis and machine intelligence* 20.2 (1998), pp. 113–125.

[6] Vera Dugina, Ingrid Zwaenepoel, Giulio Gabbiani, Sophie Clément, Christine Chaponnier. “β- and γ-cytoplasmic actins display distinct distribution and functional diversity”. In: *J. Cell. Sci.* 122 (2009), pp. 2980–2988.
