# Supplementary material for: Cytosolic actin isoforms form networks with different rheological properties that indicate specific biological function
Source: Nat Commun. 2023 Dec 2;14:7989. doi: 10.1038/s41467-023-43653-w (PMC10693642; doi:10.1038/s41467-023-43653-w)
Supplement: Supplementary file 3 — Reporting Summary [file 41467_2023_43653_MOESM3_ESM.pdf]

nature portfolio

Corresponding author(s): Andreas Janshoff

Last updated by author(s): Oct 27, 2023

Reporting Summary

Nature Portfolio wishes to improve the reproducibility of the work that we publish. This form provides structure for consistency and transparency in reporting. For further information on Nature Portfolio policies, see our Editorial Policies and the Editorial Policy Checklist.

Statistics

For all statistical analyses, confirm that the following items are present in the figure legend, table legend, main text, or Methods section.

n/a	Confirmed	
☐	☒	The exact sample size (*n*) for each experimental group/condition, given as a discrete number and unit of measurement
☐	☒	A statement on whether measurements were taken from distinct samples or whether the same sample was measured repeatedly
☐	☒	The statistical test(s) used AND whether they are one- or two-sided<br>*Only common tests should be described solely by name; describe more complex techniques in the Methods section.*
☒	☐	A description of all covariates tested
☒	☐	A description of any assumptions or corrections, such as tests of normality and adjustment for multiple comparisons
☐	☒	A full description of the statistical parameters including central tendency (e.g. means) or other basic estimates (e.g. regression coefficient) AND variation (e.g. standard deviation) or associated estimates of uncertainty (e.g. confidence intervals)
☒	☐	For null hypothesis testing, the test statistic (e.g. *F*, *t*, *r*) with confidence intervals, effect sizes, degrees of freedom and *P* value noted<br>*Give P values as exact values whenever suitable.*
☒	☐	For Bayesian analysis, information on the choice of priors and Markov chain Monte Carlo settings
☒	☐	For hierarchical and complex designs, identification of the appropriate level for tests and full reporting of outcomes
☒	☐	Estimates of effect sizes (e.g. Cohen's *d*, Pearson's *r*), indicating how they were calculated

Our web collection on statistics for biologists contains articles on many of the points above.

Software and code

Policy information about availability of computer code

Data collection	AFS Lumicks Holographic video particle tracking software AFS-Tracking-G2-v1.1.5, Olympus image acquisition software FV10-ASW 04.02.01.20, Image J 1.44, Matlab 2020b
Data analysis	All used algorithms are published and cited properly. The custom written code (in Matlab 2020b and Python 3.10) can be downloaded from gro.data doi:10.25625/OT6D7M

For manuscripts utilizing custom algorithms or software that are central to the research but not yet described in published literature, software must be made available to editors and reviewers. We strongly encourage code deposition in a community repository (e.g. GitHub). See the Nature Portfolio guidelines for submitting code & software for further information.

Data

Policy information about availability of data

All manuscripts must include a data availability statement. This statement should provide the following information, where applicable:

- Accession codes, unique identifiers, or web links for publicly available datasets
- A description of any restrictions on data availability
- For clinical datasets or third party data, please ensure that the statement adheres to our policy

The authors declare that the data of this study are available within the paper, its supplementary information files and at gro.data doi:10.25625/OT6D7M.

Data availability statement:
Source data are provided with this paper. Additional data used in this study are deposited at gro.data.[108]

[108] Janshoff, A.: Replication Data For: Cytosolic Actin Isoforms Form Networks with Different Rheological Properties that Indicate Specific Biological Function. https://doi.org/10.25625/OT6D7M

Research involving human participants, their data, or biological material

Policy information about studies with human participants or human data. See also policy information about sex, gender (identity/presentation), and sexual orientation and race, ethnicity and racism.

Reporting on sex and gender	n.a.
Reporting on race, ethnicity, or other socially relevant groupings	n.a.
Population characteristics	n.a.
Recruitment	n.a.
Ethics oversight	n.a.

Note that full information on the approval of the study protocol must also be provided in the manuscript.

Field-specific reporting

Please select the one below that is the best fit for your research. If you are not sure, read the appropriate sections before making your selection.

☒ Life sciences ☐ Behavioural & social sciences ☐ Ecological, evolutionary & environmental sciences

For a reference copy of the document with all sections, see nature.com/documents/nr-reporting-summary-flat.pdf

Life sciences study design

All studies must disclose on these points even when the disclosure is negative.

Sample size	We chose sample sizes to yield sufficient numbers of counts, based on standards of the field. No sample size calculation was performed.
Data exclusions	All data were included, only unsuccessful preparations of actin networks being omitted from the analysis.
Replication	To confirm the data's reproducibility, multiple researchers conducted experiments using various sample preparations on different days, incorporating additional technical replicates and preparing samples from multiple different batches of protein monomers. Inclusion of replicated data was unbiased in regards to previous results.
Randomization	Not applicable for our experimental design as reconstituted networks were prepared from multiple independent preparations by multiple researchers (3) and from multiple batches of protein monomers. This is standard in the field of biophysics with reconstituted systems. The precise number of preparations is given in the supplementary information. We could use standard regression analysis to obtain unbiased results.
Blinding	Blinding was unnecessary as there is no potential for bias in our measurements and data analysis.

Reporting for specific materials, systems and methods

We require information from authors about some types of materials, experimental systems and methods used in many studies. Here, indicate whether each material, system or method listed is relevant to your study. If you are not sure if a list item applies to your research, read the appropriate section before selecting a response.

Materials & experimental systems

n/a	Involved in the study
☐	☒ Antibodies
☐	☒ Eukaryotic cell lines
☒	☐ Palaeontology and archaeology
☒	☐ Animals and other organisms
☒	☐ Clinical data
☒	☐ Dual use research of concern
☒	☐ Plants

Methods

n/a	Involved in the study
☒	☐ ChIP-seq
☒	☐ Flow cytometry
☒	☐ MRI-based neuroimaging

Antibodies

Antibodies used	Primary antibodies: anti-beta-actin antibody, clone SP124, cat no. SAB5500001; anti-gamma-actin antibody, clone 2A3, cat no. MABT824 were purchased from Sigma-Aldrich Chemie GmbH (Taufkirchen, Germany)<br>Secondary antibodies: Goat anti-Rabbit IgG (H+L) Alexa Fluor™ 488, cat no. A-11008; Goat anti-Mouse IgG (H+L) Alexa Fluor™ 546, cat no. A-11003 were purchased from Life Technologies GmbH (Darmstadt, Germany)
Validation	anti-beta-actin antibody: tested (by the manufacturer) for reactivity in humans, species homologies predicted for chicken, mouse, rat, bovine, rabbit, pig, dog, hamster, frog. Flow cytometric analysis of Monoconal Rabbit Anti-Actin-beta antibody in HeLa compare to negative control of Rabbit IgG was performed (https://www.sigmaaldrich.com/DE/de/product/sigma/sab5500001) as well as immunoblotting analysis of Actin-beta in Raji cell lysate with Actin-beta antibody. 0.1 ml rabbit monoclonal antibody purified by protein A/G in PBS/1% BSA buffer pH 7.6 with less than 0.1% sodium azide.<br><br>anti-gamma-actin: tested (by the manufacturer) for reactivity in mouse, rat, human, chicken, species homologies predicted for most mammals. Evaluated by Western Blotting in HeLa cell lysate using 0.5 µg/mL of this antibody detected gamma-Actin/ACTG1 in 10 µg of HeLa cell lysate. Clone 2A3 detected BSA conjugated with cytoplasmic gamma-actin N-terminal peptide, but not BSA conjugated with N-terminal peptides derived from the other 5 actin types (Dugina, V., et al. (2009). J. Cell Sci. 122(Pt 16):2980-2988).

Eukaryotic cell lines

Policy information about cell lines and Sex and Gender in Research

Cell line source(s)	MDCK II: source: dog, Canine Cocker Spaniel Kidney, ECACC (Nr. 00062107)<br>SK-OV-3: source: human white female, ATCC No. HTB-77
Authentication	MDCK II: Authentication: via ECACC and morphology<br>SK-OV-3: Validation: ATCC and morphology
Mycoplasma contamination	MDCK II and SK-OV-3: Mycoplasma contamination: regular pcr testing confirmed cell line to be negative for mycoplasma.
Commonly misidentified lines (See ICLAC register)	commonly misidentified: none
